# P-1357. Comparative In Vitro Activity of Sulbactam-Durlobactam and Ampicillin-Sulbactam Against A. baumannii with and without PBP3 Mutation

**DOI:** 10.1093/ofid/ofaf695.1544

**Published:** 2026-01-11

**Authors:** Thomas Lavoie, Katie Daffinee, Kerry L LaPlante

**Affiliations:** Infectious Diseases Research Program, Providence Veterans Affairs Medical Center, Providence, RI; College of Pharmacy, University of Rhode Island, Kingston, RI, Cranston, RI; Infectious Diseases Research Program, Providence Veterans Affairs Medical Center, Providence, Rhode Island; 1. Infectious Diseases Research Program, Providence Veterans Affairs Medical Center, Providence, RI, United States 2. Center of Innovation in Long-Term Support Services, Providence Veterans Affairs Medical Center, Providence, RI, United States 3. College of Pharmacy, University of Rhode Island, Kingston, RI, United States 5. Warren Alpert Medical School of Brown University, Division of Infectious Diseases, Providence, RI, Kingston, RI

## Abstract

**Background:**

Treatment options for *Acinetobacter baumannii* are limited. The Infectious Diseases Society of America (IDSA) currently recommends the use of sulbactam-containing regimens whenever possible. We modeled serum concentrations of sulbactam-durlobactam (SUL-DUR), ampicillin-sulbactam (AMP-SUL), and meropenem (MER) to assist with combination treatment selection.

**Figure:**
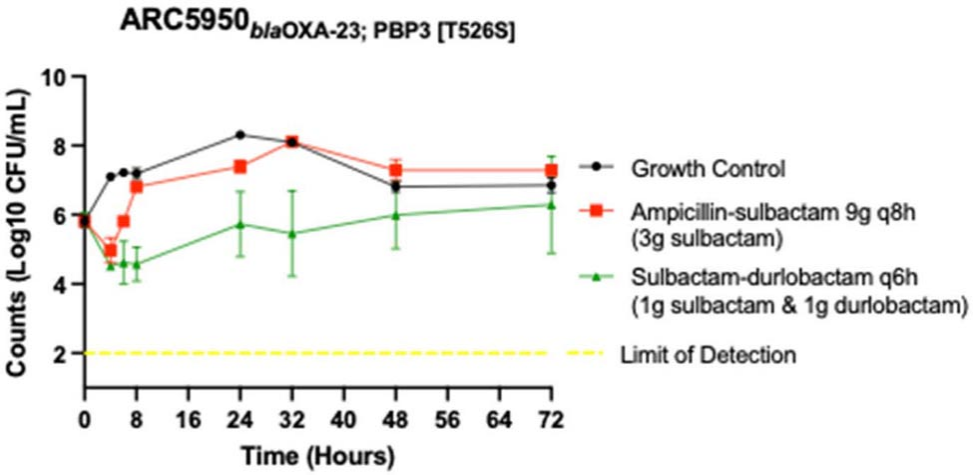
Sulbactam-durlobactam and ampicillin-sulbactam dosing simulating humanized serum concentrations at steady state against carbapenem-resistant A. baumannii with a PBP3 mutation

**Figure:**
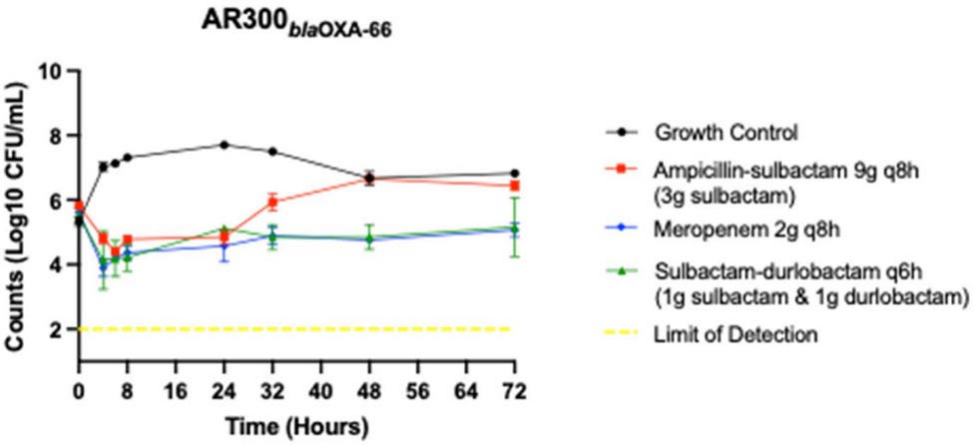
Sulbactam-durlobactam, ampicillin-sulbactam, and meropenem dosing simulating humanized serum concentrations at steady state against carbapenem-susceptible A. baumannii

**Methods:**

Two *A. baumannii* isolates were evaluated, one carbapenem-susceptible (AR300) and, one carbapenem-resistant (ARC5950) with a penicillin binding protein-3 (PBP3) mutation. We conducted one compartment in vitro pharmacodynamic models using a starting inoculum of 6-log_10_ CFU/mL, simulating monotherapies with SUL-DUR or AMP-SUL with inflow media set to the respective antibiotic half-life. We additionally ran MER treatment models against the carbapenem-susceptible strain. Models were run in duplicate, simulating humanized serum concentrations targets selected based on free drug concentrations achievable in patients using 2024 IDSA dosing recommendations. Models were sampled for CFU/mL counts at 0, 4, 6, 8, 24, 32, 48, and 72h. Susceptibility changes were evaluated by E-test every 24h and compared to 0h.

**Results:**

Against AR300, all agents demonstrated initial bacteriostatic activity after 6h. Minimum inhibitory concentration (MIC) shifts were detected as early as 24h with AMP-SUL treatment and coincided with bacterial regrowth above the initial inoculum. There were no SUL-DUR MIC shifts seen for this isolate in any SUL-DUR or AMP-SUL treated models. Antibacterial activity was similar for both MER and SUL-DUR monotherapies through 72h. Against ARC5950, only SUL-DUR treatment produced a 1-log_10_ CFU reduction. Bacterial regrowth did occur with SUL-DUR treatment, accompanied by an increased MIC in one of the two models, and exceeded the initial inoculum after 48h of treatment. AMP-SUL monotherapy produced a slight initial reduction in colony count, however, after 6h, regrowth surpassed the starting inoculum. Treatment with AMP-SUL did not correspond with elevated SUL-DUR MICs.

**Conclusion:**

SUL-DUR monotherapy was active against carbapenem-resistant *A. baumannii* with a PBP3 mutation and displayed similar activity to meropenem against carbapenem-susceptible *A. baumannii* with no MIC shifts.

**Disclosures:**

Thomas Lavoie, PharmD, innoviva: Grant/Research Support|innoviva: Co-investogator|melinta: Grant/Research Support|pfizer: Grant/Research Support|shionogi: Grant/Research Support Kerry L. LaPlante, Pharm.D., FCCP, FIDSA, FIDP, Abbvie: Advisor/Consultant|Abbvie: Grant/Research Support|Innoviva: Advisor/Consultant|Innoviva: Grant/Research Support|Melinta: Grant/Research Support|Pfizer: Grant/Research Support|Shionogi: Advisor/Consultant|Shionogi: Grant/Research Support

